# Effects of transcutaneous auricular vagus nerve stimulation on patients with post-stroke insomnia: a randomized controlled trial protocol

**DOI:** 10.3389/fpsyt.2026.1870968

**Published:** 2026-06-29

**Authors:** Jifei Sun, Siyan Chen, Chenjie Ma, Hongwei Liu, Yuan Zhou, Haoran Gao, Kexin Ma, Xiaojian Zhang, Xue Xiao

**Affiliations:** 1 Shunyi Hospital, Beijing Traditional Chinese Medicine Hospital, Beijing, China; 2 Dongzhimen Hospital, Beijing University of Chinese Medicine, Beijing, China; 3 Eye Hospital, China Academy of Chinese Medical Sciences, Beijing, China; 4 Department of Psychiatry and Psychology, Beijing Tsinghua Changgung Hospital, Beijing, China

**Keywords:** multimodal magnetic resonance imaging, neuromodulation, post-stroke insomnia, transcutaneous auricular vagus nerve stimulation, trial protocol

## Abstract

**Background:**

Post-stroke insomnia (PSI) is one of the most common and difficult-to-treat complications following stroke, with a prevalence exceeding 50%, and is an independent risk factor for stroke recurrence and secondary anxiety and depressive disorders. Current first-line strategies, namely sedative-hypnotics and cognitive behavioral therapy for insomnia, are constrained by the risks of tolerance, dependence, and cognitive side effects, as well as by limited medical resources and poor patient adherence. Transcutaneous auricular vagus nerve stimulation (taVNS), a non-invasive neuromodulation technique, has demonstrated therapeutic potential in primary insomnia and post-stroke depression. However, evidence for taVNS in PSI remains limited to a few isolated case reports; high-quality randomized controlled trials are lacking, and its underlying central nervous system mechanisms have not been characterized. This protocol is designed to address these gaps.

**Methods/design:**

This single-blind randomized controlled trial will enroll 48 patients with PSI, randomly assigned in a 1:1 ratio to a taVNS group or a sham-taVNS group. Both groups will receive the assigned intervention once daily, five days per week, for four weeks, in addition to standard post-stroke basic treatment. The primary outcomes are multimodal magnetic resonance imaging (MRI) indicators acquired before and after the 4-week intervention, including resting-state functional MRI (amplitude of low-frequency fluctuation, fractional amplitude of low-frequency fluctuation, regional homogeneity, and functional connectivity), three-dimensional T1-weighted structural metrics (gray matter volume, cortical thickness, and cortical surface area), and arterial-spin-labeling cerebral blood flow. The secondary outcomes are clinical efficacy scales, namely the Pittsburgh Sleep Quality Index, the National Institutes of Health Stroke Scale, the Activities of Daily Living scale, the 17-item Hamilton Depression Rating Scale, and the 14-item Hamilton Anxiety Rating Scale, assessed at baseline, week 2, and week 4. Between- and within-group differences will be examined using two-sample and paired t-tests with Gaussian random field correction for imaging data and repeated-measures analysis of variance for clinical data, and Pearson or Spearman correlation analyses will relate neuroimaging changes to clinical improvement.

**Discussion:**

By integrating brain function, brain structure, and cerebral perfusion through multimodal MRI, this study aims to evaluate the clinical efficacy of taVNS in patients with PSI and to elucidate its underlying central nervous system mechanisms. The main limitations are a relatively small sample size and a single-blind design, which may limit the generalizability of the findings and introduce a degree of implementation bias. Nevertheless, the findings will provide evidence-based support for the clinical application of taVNS in PSI and lay the foundation for the development of individualized neuromodulation strategies for post-stroke sleep disorders.

**Clinical trial registration:**

http://itmctr.ccebtcm.org.cn/, identifier ITMCTR2025002545.

## Introduction

Post-Stroke Insomnia (PSI) is one of the highly prevalent and intractable complications following stroke, with a prevalence rate of over 50% (1). Recent meta-analyses have further underscored the magnitude of this problem. A pooled analysis of 26 studies comprising nearly 500,000 stroke patients estimated the prevalence of post-stroke insomnia at approximately 47%, with a comparable rate of 47.21% in patients with ischemic stroke or transient ischemic attack (2). Consistently, a meta-analysis based on the Pittsburgh Sleep Quality Index reported that about 55% of stroke survivors experience poor sleep quality, with a pooled mean score of 8.12 (3). These converging estimates confirm that PSI affects roughly half of stroke survivors, far exceeding the prevalence of insomnia in the general population. Its clinical features mainly manifest as difficulty falling asleep, difficulty maintaining sleep, and circadian rhythm disruption (4). Persistent sleep deprivation not only severely reduces patients’ subjective sleep quality but also significantly impairs their cognitive function and daytime social function (5). Crucially, PSI has been identified as an independent risk factor for stroke recurrence and secondary anxiety disorders (6). Beyond its direct effects on sleep, PSI delays neurological and motor rehabilitation, prolongs hospitalization, increases caregiver burden, and is associated with poorer long-term functional recovery, thereby imposing a substantial clinical and socioeconomic burden. Therefore, in-depth exploration of effective intervention strategies for PSI holds significant clinical importance.

Current therapeutic strategies for PSI include pharmacological interventions and Cognitive Behavioral Therapy for Insomnia (CBT-I) (7). However, their clinical limitations are becoming increasingly evident. Most sedative-hypnotics not only carry risks of tolerance and dependence but may also exacerbate cognitive impairment and increase the risk of falls in stroke patients, thereby adversely affecting prognosis (8). Although CBT-I is recommended by clinical guidelines, its application is hindered by a lack of medical resources and high implementation barriers, resulting in poor patient adherence and an inability to meet the substantial clinical demand (9). Therefore, developing a novel non-pharmacological intervention that is both safe and convenient has emerged as a critical issue requiring urgent resolution in this field.

Transcutaneous auricular vagus nerve stimulation (taVNS) is an emerging non-invasive neuromodulation technique (10). Compared to traditional invasive Vagus Nerve Stimulation (iVNS), taVNS avoids surgical trauma and associated risks, offering higher safety and tolerability (11). Previous studies have confirmed the efficacy of taVNS in treating conditions such as depression (12, 13) and epilepsy (14). In the field of sleep and stroke complications, our team’s prior research has found that taVNS can effectively treat primary insomnia (15–17). Additionally, existing literature supports its role in improving post-stroke depression (18). However, research on taVNS for the treatment of PSI is still in its infancy, with only a few case reports suggesting its potential clinical efficacy (19). There remains a lack of high-quality Randomized Controlled Trials (RCTs) to provide robust evidence-based medical support. Three core gaps therefore remain: the efficacy of taVNS for PSI has not been established in adequately powered, sham-controlled trials; its therapeutic effects have not been evaluated against the multidimensional neuropathology that characterizes PSI; and the central nervous system mechanisms through which taVNS might act are still unknown.

The maintenance of the sleep-wake cycle depends on the dynamic balance at the whole-brain level, involving the precise regulation of specific neural circuits such as the thalamo-cortical loop and the reticular formation of the brainstem (20). After a stroke, ischemic injury directly disrupts these critical neuroanatomical structures and neurotransmitter pathways, leading to an imbalance between sleep drive and the arousal system (21). In recent years, with the rapid development of neuroimaging, multimodal Magnetic Resonance Imaging (MRI) technology has become a key tool for unraveling the complex neuropathological mechanisms of PSI. High-resolution T1-weighted imaging (3D-T1WI) serves as a cornerstone for assessing the integrity of brain microstructure. Techniques such as Voxel-Based Morphometry (VBM) and Surface-Based Morphometry (SBM) enable the precise quantification of key morphological indicators, including Gray Matter Volume (GMV), cortical thickness, and cortical surface area (22, 23). Previous studies have shown that chronic sleep deprivation not only leads to gray matter atrophy in brain regions related to cognition and emotion, such as the hippocampus, prefrontal cortex, and anterior cingulate cortex, but also induces widespread changes in the surface area of the sensorimotor cortex (24, 25). In PSI, these neurodegenerative changes secondary to insomnia, when superimposed on the primary stroke lesion, constitute the anatomical basis for the impeded recovery of neurological function in patients.

As an effective tool for investigating spontaneous neuronal activity by monitoring fluctuations in blood oxygen levels, resting-state functional MRI (rs-fMRI) helps assess the spontaneous evolution of Blood Oxygen Level-Dependent (BOLD) signals and abnormalities in functional connectivity within core brain regions regulating “emotion-arousal” in patients with PSI (26). Recent fMRI studies reveal that PSI is characterized by multi-dimensional spontaneous activity abnormalities, specifically within the visual processing cortex, sensorimotor cortex, and Default Mode Network (DMN) nodes (26). Furthermore, Arterial Spin Labeling (ASL) provides non-invasive, contrast-free quantification of Cerebral Blood Flow (CBF) (27). ASL effectively captures hypoperfusion in ischemic areas and sleep-related brain regions, thereby reflecting the degree of impairment in neurovascular coupling function (28).

Taken together, the pathophysiology of PSI can be conceptualized as a multidimensional process spanning structural damage, functional disruption, and hemodynamic abnormality. This framework provides a clear rationale for taVNS. As a “bottom-up” intervention, taVNS delivers afferent signals through the auricular branch of the vagus nerve to the nucleus tractus solitarius, which in turn projects to the locus coeruleus, thalamus, and limbic and prefrontal regions, that is, the same arousal- and emotion-regulating circuits that are structurally, functionally, and hemodynamically compromised in PSI. taVNS therefore has the potential to act simultaneously on all three of these dimensions, yet this possibility has not been tested. Accordingly, this study adopts a randomized controlled design that integrates 3D-T1WI structural imaging, BOLD-fMRI, and ASL perfusion to evaluate the clinical efficacy of taVNS in PSI and to characterize its effects across brain structure, neural function, and cerebral perfusion. By further examining the cumulative effects of a 4-week intervention, the study aims to clarify how taVNS drives neuroplastic remodeling and to provide a robust theoretical basis for precise clinical intervention in PSI.

## Methods

### Study design

**Figure 1:**
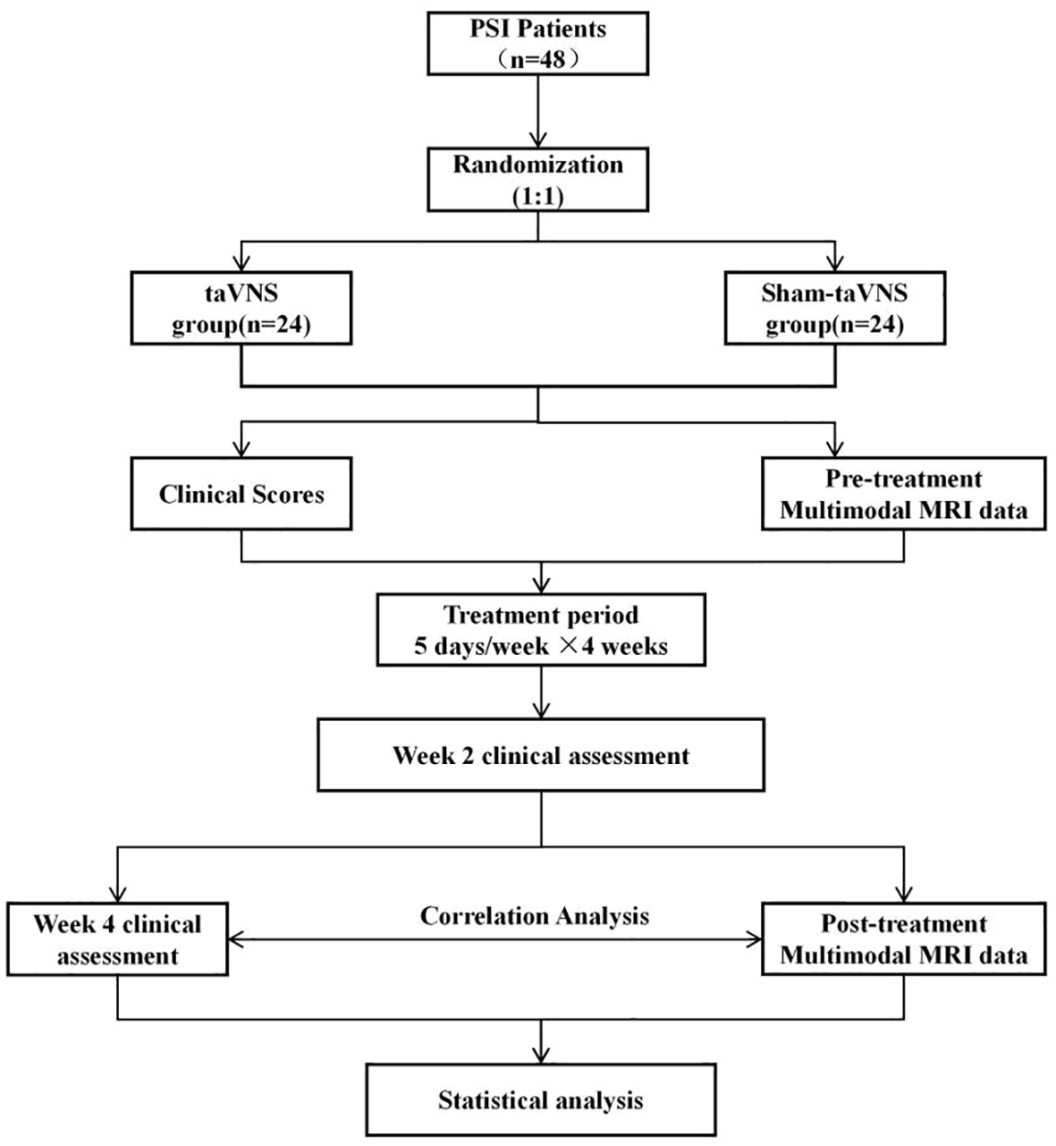
Flowchart of the trial. Clinical outcome assessments are conducted at three time points: baseline (Week 0), 2-week evaluation (Week 2), and 4-week evaluation (Week 4). PSI, Post-stroke insomnia; taVNS, transcutaneous auricular vagus nerve stimulation; MRI, Magnetic Resonance Imaging.

This is a prospective, randomized controlled clinical trial. A total of 48 patients with PSI will be screened according to strict inclusion and exclusion criteria. The protocol has been registered with the International Traditional Medicine Clinical Trial Registry (ITMCTR2025002545) and strictly follows the principles of the Declaration of Helsinki. It has been approved by the Medical Ethics Committee of Beijing Hospital of Traditional Chinese Medicine Shunyi Hospital (2025SYKY-04-03). All participants or their legal guardians are required to sign a written informed consent form prior to enrollment. **Figure 1** provides the detailed information of the study design.

### Inclusion criteria

(1) Confirmed by MRI and meeting the diagnostic criteria for ischemic cerebrovascular disease; (2) Aged between 40 and 80 years, regardless of gender; (3) Insomnia first occurring more than 2 weeks after acute stroke, with no prior history of insomnia; (4) Pittsburgh Sleep Quality Index (PSQI) score > 7; (5) Conscious and able to cooperate with all scale assessments; (6) Voluntary participation in the study, signing the informed consent form, and committing not to participate in other clinical studies during the treatment period.

### Exclusion criteria

(1) Unstable condition or recurrence of stroke during the treatment period; (2) Severe bleeding tendency, or concurrent failure of vital organs such as the heart, liver, or kidneys; (3) Insomnia caused by other definite etiologies; (4) Comorbid severe mental illness; (5) Currently participating in other clinical trials; (6) Contraindications to MRI; (7) Hemorrhagic stroke patients; (8) History of ear infection or prior vagus nerve surgery.

### Withdrawal criteria

(1) Severe adverse reactions or allergic reactions; (2) Significant disease progression.

### Sample size calculation

Currently, no precise method exists for calculating the sample size for acupuncture neuroimaging trials. Based on international reviews, a minimum of 12 cases per group is recommended (29, 30). To account for potential data loss and exclusions, this trial will enroll 24 subjects per group, totaling 48 participants.

### Blinding

To ensure methodological rigor, this trial adopts a single-blind design. Participants will be blinded to group allocation and treated in separate rooms to minimize communication between groups. To assess the effectiveness of the blinding, participants will be asked to guess their group assignment (taVNS group or sham-taVNS group) after the intervention. Due to the specific nature of the taVNS intervention, the therapists cannot be blinded. In contrast, both the outcome assessors and the statistical analysts will remain blinded to the group assignments throughout the study.

Eligible participants will be randomly assigned to the taVNS group or sham-taVNS group in a 1:1 ratio using a computer-generated random sequence. Allocation concealment will be maintained using sequentially numbered, opaque, sealed envelopes prepared by an independent researcher who is not involved in recruitment, intervention, outcome assessment, or data analysis.

### Interventions

In the taVNS group, a taVNS device will be used (Hwato, SDZ-IIB, Suzhou, China) to stimulate the auricular branch of the vagus nerve distribution area located posterior and inferior to the external auditory meatus, with clear anatomical localization. The stimulation parameters will be as follows (31, 32): (1) dense-sparse waves with a pulse frequency of 4/20 Hz; (2) current intensity of 3–14 mA, individually adjusted according to the patient’s tolerance; (3) stimulation time of 30 min per session. Patients will be placed in a comfortable position. The treatment will be administered once daily, five days per week, for a total of four weeks. These parameters were chosen on the basis of our team’s previous taVNS trials in primary insomnia and depression, in which the same dense-disperse 4/20 Hz waveform and 30-minute daily session produced clinical benefit with good safety and tolerability (15, 31, 32). The auricular branch of the vagus nerve distribution was selected as the stimulation target because this region is densely innervated by vagal afferent fibers, enabling direct, non-invasive activation of the vagal pathway. The current intensity will be individually titrated within the 3–14 mA range to the highest level that evokes a distinct but non-painful tingling sensation, so as to ensure adequate afferent activation while maintaining tolerability and minimizing the risk of adverse effects (10). The 4-week course was set to match the intervention duration shown to be effective in our prior insomnia studies and to align with the typical timescale of post-stroke neuroplastic change.

**Figure 2:**
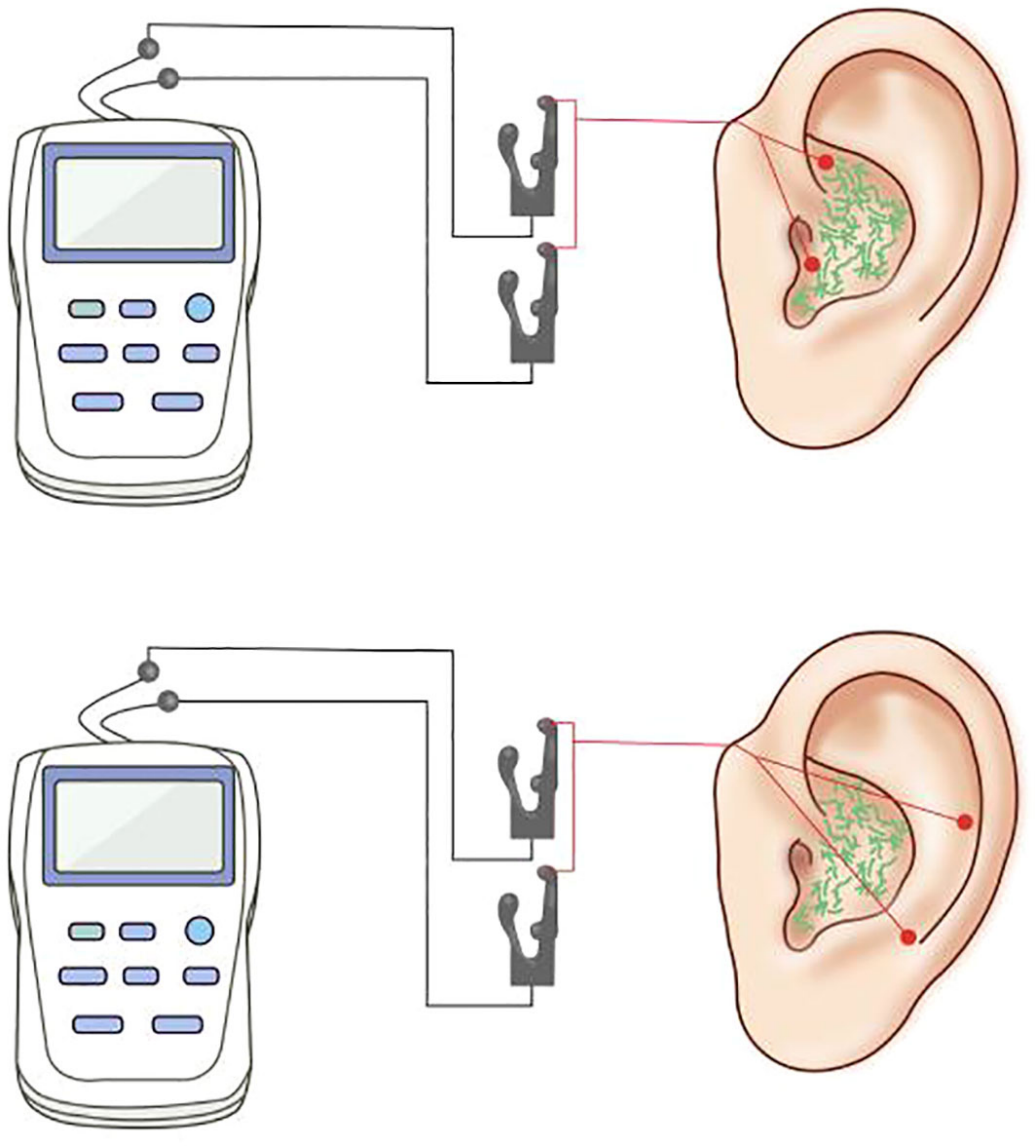
Stimulation sites for taVNS and sham taVNS. The green area indicates the auricular branch of the vagus nerve distribution.

In the sham-taVNS group, the same therapeutic device will be used, but the stimulation site will be the superior scaphoid fossa at the midpoint of the external auricular margin, an area not innervated by the vagus nerve (**Figure 2**). The selection of this site as the sham target is supported by authoritative anatomical evidence. In the classic cadaveric dissection of the human auricle, the auricular branch of the vagus nerve was found to innervate the cymba conchae (100%), the cavity of concha, and the tragus, whereas the scapha and scaphoid fossa receive their cutaneous innervation from the great auricular and auriculotemporal nerves rather than the vagus nerve (33). Accordingly, the superior scaphoid fossa lies outside the vagal innervation territory and is not expected to activate central vagal afferent pathways. Consistent with this anatomy, the scaphoid fossa has been adopted as a non-vagal sham site in previous double-blind, sham-controlled taVNS trials, in which stimulation of this region produced comparable tactile sensation without engaging the vagal pathway (34). The stimulation parameters and treatment course will be the same as those in the treatment group.

### Basic treatment

During the 4-week intervention period, all participants will receive standard basic treatment, including conventional post-stroke medication and rehabilitation training. Medication will be administered in accordance with the *Chinese Guidelines for the Diagnosis and Treatment of Acute Ischemic Stroke 2023* (35). Rehabilitation training will include functional training, routine medical care, and other standard rehabilitation procedures.

To avoid potential confounding effects on sleep outcomes and multimodal MRI indicators, sedative-hypnotic drugs will not be routinely prescribed during the study period. Estazolam will be permitted only as rescue medication when participants experience severe or intolerable insomnia, such as inability to sleep for two consecutive nights or when clinically judged necessary by the investigator. The recommended rescue dose will be estazolam 1 mg orally before bedtime. The use of rescue medication, including dosage, frequency, and timing, will be recorded in detail throughout the study. Participants will be instructed to avoid taking estazolam within 48 hours before MRI scanning and clinical assessments whenever clinically feasible.

### Multimodal MRI data acquisition

All multimodal MRI data will be acquired on a Siemens 3.0T Vida scanner at the Shunyi Hospital of Beijing Hospital of Traditional Chinese Medicine. For each participant, scanning will be performed at two time points: on the day before the first treatment session and on the day after completing the 4-week intervention. To minimize variability, all scans will be obtained using identical hardware, sequences, and acquisition parameters at both time points.

Three sequences will be acquired in a fixed order. First, resting-state functional MRI (rs-fMRI) will be obtained using a gradient-echo echo-planar imaging (GRE-EPI) sequence to capture spontaneous neural activity. During the scan, participants will be instructed to remain awake with their eyes closed, stay as still as possible, and refrain from engaging in any structured thought. Next, a three-dimensional T1-weighted image (3D-T1WI) will be acquired to provide high-resolution structural information for morphometric analysis and for spatial normalization of the functional and perfusion data. Finally, arterial spin labeling (ASL) perfusion imaging will be performed to quantify cerebral blood flow. The detailed acquisition parameters for all three sequences are summarized in **Table 1**.

**Table 1:** Acquisition parameters of the multimodal MRI sequences.

Parameter	rs-fMRI (GRE-EPI)	3D-T1WI	ASL
Repetition time, TR (ms)	2000	2530	4200
Echo time, TE (ms)	30	2.98	38.06
Flip angle, FA (°)	90	7	120
Field of view, FOV (mm)	224 × 224	256 × 256	224 × 224
Matrix	64 × 64	256 × 256	64 × 64
Slice thickness (mm)	3.5	1.0	3.5
Number of slices	32	192	46
Voxel size (mm³)	3.5 × 3.5 × 3.5	1.0 × 1.0 × 1.0	3.5 × 3.5 × 3.5
Number of time points	200	—	—
Acquisition time	6 min 46 s	6 min 3 s	7 min 21 s

rs-fMRI, resting-state functional MRI; GRE-EPI, gradient-echo echo-planar imaging; 3D-T1WI, three-dimensional T1-weighted imaging; ASL, arterial spin labeling; TR, repetition time; TE, echo time; FA, flip angle; FOV, field of view.

### Outcome measures

#### Primary outcomes

The primary outcomes include neuroimaging indicators derived from fMRI, 3D-T1WI, and ASL, specifically: The primary outcome is defined as the change in these multimodal MRI indicators from baseline to the end of the 4-week intervention; the principal comparison is the between-group difference in these changes (taVNS vs. sham-taVNS), with within-group pre-to-post changes assessed as a secondary contrast. The specific indicators are as follows:

##### fMRI

###### Amplitude of low-frequency fluctuation and fractional amplitude of low-frequency fluctuation

ALFF effectively reflects spontaneous neural activity in the cerebral cortex by calculating the average amplitude of low-frequency signal fluctuations, and is a commonly used method for analyzing signal characteristics of individual voxels or localized brain regions (36). This metric has been widely applied in studies of spontaneous brain activity under various conditions (37). fALFF is an improved analytical method based on ALFF. It effectively reduces the susceptibility of ALFF to physiological noise interference in the cerebral pool and ventricular regions, offering higher sensitivity and specificity for the detection of spontaneous brain activity (38).

###### Regional homogeneity

ReHo is based on Kendall’s coefficient of concordance and evaluates the similarity between a target voxel and its neighboring voxels over a time series on a voxel-by-voxel basis (39). This method is widely used in the analysis of changes in local spontaneous neural activity in neuropsychiatric disorders, as well as in studies of the central mechanisms of taVNS (40).

###### Functional connectivity

FC refers to the temporal synchrony between distant neurons or brain regions during rest. It is typically measured by calculating the temporal correlation coefficients of BOLD signals across different brain regions, reflecting functional integration and information exchange between brain regions (41, 42).

##### 3D-T1WI

###### Gray matter volume

GMV is one of the most widely used indicators derived from 3D-T1WI structural imaging. Through the VBM pipeline, the acquired T1-weighted images are segmented into gray matter, white matter, and cerebrospinal fluid (CSF) tissue maps, which are then spatially normalized and modulated to enable voxel-wise comparison of regional GMV across subjects (43). GMV reflects the density and integrity of neuronal cell bodies, dendrites, and synapses within a given brain region, serving as a sensitive marker for detecting neurodegenerative and neuroplastic changes (44).

###### Cortical thickness

Cortical thickness (CT) is a key morphometric indicator obtained through SBM analysis. Unlike VBM, which operates in volumetric space, SBM reconstructs the cortical surface by identifying the boundary between Gray Matter (GM) and white matter and the boundary between GM and CSF, and then calculates the shortest distance between the two surfaces at each vertex (45). CT is considered a sensitive biomarker reflecting cortical cytoarchitecture, columnar organization, and myelination status, and has been shown to be influenced by aging, neurodegeneration, and experience-dependent plasticity (46).

###### Cortical surface area

Cortical Surface Area (CSA) is another important morphometric parameter derived from SBM analysis. It measures the total area of the cortical mantle at each vertex and is closely related to the tangential expansion of cortical columns during neurodevelopment (47). Unlike cortical thickness, which primarily reflects radial growth and dendritic arborization, CSA is thought to be largely determined by the number of cortical minicolumns and is influenced by distinct genetic and environmental factors (48).

##### ASL

###### Cerebral blood flow

CBF is the core quantitative parameter obtained through arterial spin labeling imaging. It represents the volume of blood delivered to a unit mass of brain tissue per unit time and directly reflects local cerebral perfusion status and metabolic demand (49, 50). Through neurovascular coupling mechanisms, cerebral blood flow is closely associated with local neuronal activity: increased neuronal activity induces local vasodilation, leading to a corresponding increase in blood supply to meet the elevated metabolic demands of active neurons (51). Therefore, CBF serves as both a reliable surrogate marker of local cerebral metabolism and an effective indicator of the integrity of neurovascular coupling.

#### Secondary outcomes

Secondary outcome measures include efficacy-related scales, specifically the Pittsburgh Sleep Quality Index (PSQI), the National Institutes of Health Stroke Scale (NIHSS), the Activities of Daily Living (ADL) assessment scale, the 17-item Hamilton Depression Rating Scale (HAMD-17), and the 14-item Hamilton Anxiety Rating Scale (HAMA-14). These scales will be assessed once each at baseline (Week 0), Week 2, and Week 4 of treatment. The PSQI is the principal secondary endpoint, reflecting subjective sleep quality, while the NIHSS, ADL, HAMD-17, and HAMA-14 are supportive secondary endpoints capturing neurological function, daily living ability, and depressive and anxiety symptoms, respectively. For each scale, the between-group difference in the change from baseline and the time course across the three assessment points (Week 0, Week 2, and Week 4) will be evaluated. In addition, the use of rescue medication, including the number of nights and total dose of estazolam, will be recorded as an exploratory outcome.

The PSQI is used to assess sleep quality in post-stroke patients. This scale is widely used in clinical research, has good reliability and validity, and can effectively distinguish between different groups with sleep disorders (52). The NIHSS is a simple, rapid, and effective tool for assessing neurological deficits following stroke. It exhibits high intra- and inter-rater reliability and is widely used both domestically and internationally to evaluate the severity of neurological deficits and disease progression (53). The ADL assessment scale is used to evaluate the ability of post-stroke patients to live independently. It covers basic daily activities such as eating, dressing, and toileting, and can objectively reflect the level of functional recovery and the effectiveness of rehabilitation treatment (53). The HAMD-17 is used to assess the severity of depressive symptoms in post-stroke patients. It is one of the most commonly used depression assessment tools in clinical research and demonstrates good internal consistency and inter-rater reliability (54). The HAMA-14 is used to assess the severity of anxiety symptoms in post-stroke patients. It effectively reflects changes in anxiety status, possesses good reliability and validity, and is suitable for clinical efficacy assessment (55).

### Data management

All clinical data for this study will be managed using the data management system of the Shunyi Branch of Beijing Hospital of Traditional Chinese Medicine, and regular data audits will be conducted. Serious adverse events and unexpected events will be reported promptly to the Ethics Committee and regulatory authorities in accordance with regulations. Progress reports will be submitted on schedule, detailing adverse events, subject withdrawals, and protocol deviations. The principal investigator will conduct periodic cumulative reviews of all adverse events and, if necessary, convene investigator meetings to assess the risks and benefits of the study.

### Data analysis

#### Imaging data analysis

The rs-fMRI data will be preprocessed using SPM12 (https://www.fil.ion.ucl.ac.uk/spm/) and DPARSF 5.0 toolkit (56) with Matlab 2020a. Data preprocessing includes the first ten time points removed to allow for magnetization equilibrium, slice timing correction, realignment for head motion correction (subjects with head motion exceeding 2 mm in translation or 2° in rotation will be excluded), spatial normalization to Montreal Neurological Institute (MNI) space and resampled at 3 mm × 3 mm × 3 mm voxel size, spatial smoothing with a 6 mm full-width at half maximum Gaussian kernel, linear detrending, nuisance covariates regression (including 24 head motion parameters, white matter signals, and CSF signals), and band-pass filtering (0.01–0.08 Hz).

After standard preprocessing, ReHo, ALFF, and fALFF analyses will be calculated to explore regional brain activity. For FC analysis, seed regions will be defined based on brain areas showing significant differences in ALFF, fALFF, or ReHo analyses, and the mean time series of each seed region will be correlated with the time series of all other voxels across the whole brain using Pearson’s correlation coefficients, which will be subsequently transformed to z-values using Fisher’s r-to-z transformation to improve normality (57). Two-sample Student’s t-tests and paired Student’s t-tests will be conducted respectively to examine the differences in taVNS-related central responses between groups and within groups. The results will be corrected by Gaussian Random Field (GRF) Theory (voxel-level *P* < 0.005, cluster-level *P* < 0.05) (58).

Structural 3D-T1WI data processing will be performed using two complementary approaches. For VBM, the CAT12 toolbox (http://www.neuro.uni-jena.de/cat/) implemented in SPM12 will be employed. Data preprocessing includes bias-field correction, tissue segmentation into GM, White Matter (WM), and CSF maps, spatial normalization to Montreal Neurological Institute (MNI) space using the Diffeomorphic Anatomical Registration Through Exponentiated Lie Algebra (DARTEL) algorithm, modulation to preserve the total amount of tissue within each voxel, resampling at 1.5 mm × 1.5 mm × 1.5 mm voxel size, and spatial smoothing with an 8 mm Full-Width at Half Maximum (FWHM) Gaussian kernel. For SBM, cortical reconstruction and parcellation will be performed using FreeSurfer (ver. 7.2; https://surfer.nmr.mgh.harvard.edu/). The processing pipeline includes motion correction, intensity normalization, skull stripping, automated tissue segmentation, identification of the white matter and pial surfaces, cortical surface inflation and registration to a spherical atlas, and cortical parcellation based on the Desikan-Killiany atlas (59). CT will be computed as the shortest distance between the white matter and pial surfaces at each vertex, and cortical surface area (CSA) will be calculated as the total area of the cortical mantle at each vertex. Surface maps will be smoothed with a 10 mm FWHM Gaussian kernel. Two-sample Student’s t-tests and paired Student’s t-tests will be applied to examine inter-group and intra-group differences in GMV, CT, and CSA, respectively. VBM results will be corrected by GRF Theory (voxel-level *P* < 0.005, cluster-level *P* < 0.05), and SBM results will be corrected using the Monte Carlo cluster-wise correction method (cluster-forming threshold *P* < 0.001, cluster-level *P* < 0.05) (60).

ASL data processing will be performed using SPM12 and the ASLtbx toolbox (https://www.nitrc.org/projects/asltbx/). Preprocessing includes motion correction of the ASL time series, co-registration to the individual 3D-T1WI structural image, pairwise subtraction between labeled and control images to generate perfusion-weighted images, and quantitative CBF map calculation using the single-compartment model in accordance with the consensus recommendations (61). The CBF maps will then be spatially normalized to MNI space using the transformation parameters derived from the structural image normalization, resampled at 2 mm × 2 mm × 2 mm voxel size, and spatially smoothed with a 6 mm FWHM Gaussian kernel. Two-sample Student’s t-tests and paired Student’s t-tests will be conducted to examine the between-group and within-group differences in regional CBF, respectively. The results will be corrected by GRF Theory (voxel-level *P* < 0.005, cluster-level *P* < 0.05). Across all imaging modalities, these thresholds were applied to control the family-wise error rate; GRF correction was used for the voxel-wise analyses (rs-fMRI, VBM, and ASL), whereas the Monte Carlo cluster-wise method was adopted for the surface-based analyses (CT and CSA) because it is better suited to the geometry of the cortical surface. These criteria follow widely adopted neuroimaging conventions, balancing protection against false positives with sensitivity to true taVNS-related effects.

Additionally, Pearson’s correlation analyses will be performed to explore the relationships between changes in neuroimaging indicators (ALFF, fALFF, ReHo, FC, GMV, CT, CSA, and CBF) and changes in clinical outcome measures (PSQI, NIHSS, ADL, HAMD-17, and HAMA-14), with statistical significance set at *P* < 0.05 (Bonferroni-corrected). For these brain-behavior correlations, the mean values of each neuroimaging metric will first be extracted from the clusters showing significant between- or within-group differences in the preceding voxel- or vertex-wise analyses; these extracted values, rather than whole-brain maps, will then be correlated with the corresponding changes in clinical scale scores. Pearson’s correlation will be applied when both variables satisfy the assumptions of normality and linearity, and Spearman’s rank correlation will be used otherwise. The taVNS and sham-taVNS groups will be analyzed separately, and the Bonferroni method will be used to correct for the number of correlation tests performed.

#### Clinical data analysis

All data in this study will be analyzed using SPSS 26.0 software (IBM Corp., Armonk, NY, USA). Continuous data following a normal distribution are expressed as mean ± standard deviation (Mean ± SD). Intergroup comparisons will be performed using the independent samples t-test, while pre- and post-treatment comparisons within groups will be performed using the paired t-test. Continuous data not following a normal distribution are expressed as median and interquartile range [M(P25, P75)], and intergroup comparisons will be performed using the Mann-Whitney U test. For dynamic observational data involving multiple time points, repeated measures ANOVA will be used. Categorical data will be described using frequencies and percentages (%). Intergroup comparisons for ordinal categorical data will be performed using the chi-square (χ²) test or Fisher’s exact test, while for nominal categorical data, the Kruskal-Wallis H rank-sum test will be used. Furthermore, for the correlation between clinical symptom scales and multimodal MRI indices, Pearson or Spearman correlation analysis will be used, depending on whether the data meet the assumptions of normal distribution and linearity. All statistical tests will be two-sided, and *P* < 0.05 will be considered statistically significant.

### Patient safety

Any Adverse Events (AEs) or Serious Adverse Events (SAEs) occurring during the intervention period, regardless of whether the investigator considers them to be related to the taVNS intervention, will be accurately and thoroughly documented in the case report form to allow for a rigorous assessment of their relevance. For common mild adverse events associated with taVNS, such as erythema of the auricular region, tingling or burning sensations, contact dermatitis, and occasional dizziness, the investigator will immediately implement symptomatic management, such as suspending stimulation, adjusting parameters, or providing local skin care. Given that the study participants are post-stroke insomnia patients with underlying cardiovascular and cerebrovascular risk factors, if serious adverse events such as stroke recurrence, severe heart failure, or acute organ injury occur, the investigator will immediately discontinue the intervention and promptly transport the patient to the emergency department or a neurology specialty unit for emergency treatment; any related medical expenses incurred will be borne by the research team. Furthermore, any serious adverse event must be urgently reported to the hospital’s Institutional Review Board (IRB) and relevant administrative departments within 24 hours of becoming known, in strict accordance with standardized procedures.

## Discussion

To our knowledge, this is one of the first clinical trial protocols to investigate taVNS for the treatment of PSI. This study will utilize multimodal MRI to examine the effects of taVNS on brain function, brain structure, and cerebral blood flow. By combining these findings with clinical data, we aim to evaluate the clinical efficacy of taVNS in treating PSI and, in doing so, gain preliminary insights into the underlying mechanisms of central nervous system plasticity.

As a commonly used neuromodulation technique in current research on psychiatric and neurological disorders, taVNS has established a solid research foundation in post-stroke conditions such as motor dysfunction (62), post-stroke depression (18), and post-stroke dysphagia (63). However, current research combining multimodal MRI to investigate the role of taVNS in post-stroke conditions remains relatively scarce. Therefore, conducting research on the mechanisms underlying the comorbidity of neurological and psychiatric disorders using taVNS holds significant practical importance.

This study aims to comprehensively employ fMRI, T1WI, and ASL techniques to analyze relevant imaging parameters, thereby investigating the associations among brain functional activity, structural characteristics, and changes in cerebral blood flow, with the goal of providing further evidence for the potential mechanisms underlying taVNS treatment for PSI. rs-fMRI can detect spontaneous neural activity in the brain during a task-free state and identify spatially distributed networks with temporal synchrony, thereby reflecting the topological properties of resting-state brain networks (64). Recent studies have shown that abnormal changes in resting-state networks are closely associated with the progression and severity of the disease (65, 66). Abnormal brain structure also plays a critical role in the onset and maintenance of insomnia. 3D-T1WI can quantitatively assess gray matter morphological features, such as cortical volume and thickness (67). ASL, as a non-invasive perfusion imaging technique, accurately reflects changes in cerebral blood flow, aiding in the localization of functional and perfusion abnormalities in brain regions associated with insomnia (68, 69). Further correlation analysis between clinical indicators such as the PSQI and imaging parameters obtained from rs-fMRI, T1, and ASL will help reveal the intrinsic links between improvements in sleep quality and changes in brain function, structure, and perfusion following taVNS intervention.

Such integrated analyses are expected to identify specific neuroimaging biomarkers that can not only predict treatment responsiveness but also clarify the central mechanisms through which taVNS exerts its therapeutic effects. For example, the association between enhanced functional connectivity in the DMN and reduced PSQI scores may suggest that taVNS exerts its sleep-promoting effects by modulating large-scale neural networks involved in self-referential processing and arousal regulation (70). Similarly, the correlation between increased cerebral blood flow in the thalamus and prefrontal regions and improved sleep maintenance may indicate that the restoration of neurovascular coupling is one of its key mechanisms of action (71). Building on these prior mechanistic findings, we can make several specific predictions regarding the brain regions most likely to be modulated by taVNS in PSI. At the functional level, given that taVNS has been shown to normalize spontaneous activity and to enhance connectivity within the default mode network, thalamus, and prefrontal cortex in primary insomnia and depression (17, 40, 70), we anticipate that the taVNS group will show increased ALFF/fALFF and ReHo in the prefrontal cortex and precuneus, together with strengthened thalamo-cortical and intra-DMN functional connectivity. At the structural level, because chronic sleep deprivation is associated with gray matter atrophy in the hippocampus, prefrontal cortex, and anterior cingulate cortex (24, 25), a 4-week intervention may produce measurable increases, or attenuated loss, of gray matter volume and cortical thickness in these emotion- and cognition-related regions. At the perfusion level, taVNS is expected to increase cerebral blood flow in the thalamus and prefrontal cortex, reflecting restored neurovascular coupling in hypoperfused, sleep-regulating areas. We further hypothesize that these regional changes will correlate with clinical improvement, such that greater enhancement of prefrontal-thalamic function and perfusion is accompanied by larger reductions in PSQI and HAMD/HAMA scores. Specifying these candidate regions and directional hypotheses in advance strengthens the forward-looking value of the protocol and provides concrete, testable targets for the planned analyses.

In addition to elucidating the neural correlates of clinical improvement, the multimodal MRI approach employed in this study offers the unique advantage of capturing the multidimensional characteristics of taVNS-induced neuroplasticity. By integrating structural, functional, and perfusion measures, this approach enables a more comprehensive understanding of how peripheral neural modulation translates into reorganization of the central nervous system. It can be inferred that taVNS exerts synchronous effects on cortical excitability, regional cerebral blood flow, and gray matter microstructure via its afferent projections to the nucleus tractus solitarius. Elucidating these interrelated mechanisms will not only advance our fundamental understanding of vagal neural circuits but also provide a theoretical basis for optimizing stimulation protocols and achieving personalized treatment.

Although this study has many strengths, there are several limitations that warrant mention. First, the planned sample size is relatively small; although it meets the recommended minimum for acupuncture neuroimaging trials, it may limit the statistical power and the generalizability of the findings. Second, this is a single-center study, which may further constrain the representativeness of the sample and the external validity of the results. Third, the intervention period is relatively short (4 weeks), and no follow-up assessment is planned, so the long-term efficacy and the durability of the taVNS effect cannot be determined. Fourth, although the single-blind design is methodologically sound, it may introduce a degree of implementation bias. To address these limitations, future studies should conduct larger-scale, multicenter randomized controlled trials with extended follow-up periods to further validate the findings of this study and the long-term durability of the taVNS effect. Furthermore, future studies should consider incorporating objective measurement indicators such as polysomnography and integrating multiple neuroimaging modalities, including diffusion tensor imaging and magnetic resonance spectroscopy. This will help elucidate the microstructural and metabolic basis of the neuroplastic changes induced by taVNS in patients with PSI.

In conclusion, the clinical management of PSI continues to present significant challenges. As an economical, non-invasive, and promising “bottom-up” neuromodulation technique, taVNS offers new possibilities for clinical intervention. This study protocol aims to further elucidate the central nervous system mechanisms underlying the therapeutic effects of taVNS in PSI. The findings will not only deepen our understanding of this therapy but also provide a crucial basis for advancing treatment strategies for patients with PSI.
